# Efficiency and safety of a guidewire-less approach to superselective transarterial microcatheter procedures in hepatic cancer interventions

**DOI:** 10.1186/s12885-026-15907-5

**Published:** 2026-03-25

**Authors:** Pi-Yi Chang, Wei-Ru Chiou

**Affiliations:** 1 Department of Medical Imaging, Taichung Veterans General Hospital, Taichung, 407 Taiwan; 2 Department of Post-Baccalaureate Medicine, College of Medicine, National Chung Hsing University, Taichung, 402 Taiwan; 3 Division of Cardiology, Taitung MacKay Memorial Hospital, Taitung, 950 Taiwan; 4 Department of Medicine, MacKay Medical University, New Taipei City, 252 Taiwan; 5 College of Science and Engineering, National Taitung University, Taitung, 950 Taiwan

**Keywords:** Hepatocellular carcinoma, Guidewire-less, Procedure time-associated radiation dose, Transcatheter Arterial Chemoembolization (TACE)

## Abstract

**Background:**

No study of patients with hepatocellular carcinoma (HCC) has compared the procedure time of transcatheter arterial chemoembolization (TACE) using guidewire-less and guidewire techniques. The present study aimed to further compare the procedure time and radiation exposure of the “*W*ireless *A*ngiographic *VE*ssel *S*election” (WAVES) technique, hybrid procedure and conventional guidewire TACE.

**Methods:**

Procedure time and radiation dose were compared between patients with HCC treated with the WAVES (*n* = 125) or conventional TACE (*n* = 59). Among WAVES group, 36 patients were switched to conventional procedure if the desired location could not be reached after 3 min of initiated WAVES procedure (hybrid group). In the per-treatment analysis, procedure and radiation dose were also compared between the 3 group.

**Results:**

WAVES procedure (β = -13.694, 95% confidence interval [CI]: -18.215, -9.173, *p* < 0.001) was an independent predictor of shorter procedure time compared to a conventional procedure. A longer procedure time was an independent predictor of higher radiation dose (β = 14.216, 95% CI: 12.654, 15.777, *p* < 0.001). After stratifying for vessel type (moderate difficulty [MD] and high difficulty [HD]), an association between WAVES and shorter procedure time was also found in both MD (WAVES vs. conventional: β = -21.085, 95% CI: -28.799, -13.371, *p* < 0.001) and HD (WAVES vs. conventional: β = -10.784, 95% CI: -16.644, -4.924, *p* < 0.001) subgroups. An association between longer procedure time and higher radiation dose was also found in both MD (β = 14.938, 95% CI: 12.694, 17.183, *p* < 0.001) and HD (β = 13.617, 95% CI: 11.293, 15.942, *p* < 0.001) subgroups. In the per-treatment analysis, the similar associations between WAVES procedure and procedure time and radiation dose as compared to conventional procedure. In addition, hybrid procedure (β = -12.202, 95% CI: -17.697, -6.708, *p* < 0.001) was as an independent predictor of shorter procedure time compared to a conventional procedure. A longer procedure time was an independent predictor of higher radiation dose (β = 14.389, 95% CI: 12.962, 15.816, *p* < 0.001). After stratifying for vessel type, an association between hybrid procedure and shorter procedure time was only found in the MD subgroup (hybrid vs. conventional: β = -19.771, 95% CI: -34.280, -5.263, *p* = 0.009), but this association was not observed in the HD subgroup (hybrid vs. conventional: β = -6.595, 95% CI: -15.894, 2.705, *p* = 0.160). An association between longer procedure time and higher radiation dose was also found in both MD (β = 15.046, 95% CI: 13.097, 16.995, *p* < 0.001) and HD (β = 13.869, 95% CI: 11.719, 16.018, *p* < 0.001) subgroups.

**Conclusion:**

The use of WAVES or hybrid procedure is associated with shorter procedure duration in patients with HCC undergoing TACE, which reduces radiation dose. It may be deployed as a first-line approach for MD rather than HD vessels.

**Supplementary Information:**

The online version contains supplementary material available at 10.1186/s12885-026-15907-5.

## Background

 The advent of steerable microcatheters with tips smaller than 2.4 Fr has opened the possibility of interventional procedures and techniques that do not require guidewires, including superselective transcatheter arterial chemoembolization (TACE). A small number of studies have explored the use of guidewire-less techniques in various contexts [1–9], and results suggest that the primary advantages of guidewire-less techniques are decreased risk of vessel perforation and intimal injury, and time saved from eliminating micro-guidewire insertion, withdrawal, and re-insertion. Nevertheless, most studies have focused on the feasibility and safety of a specific procedure [6–8], or make general comparisons of a variety of procedures. Few studies have performed direct comparisons of the procedure time of TACE using guidewire-less techniques and guidewire techniques [2], and none have studied patients with hepatocellular carcinoma (HCC). Detection of tumor feeders using intraprocedural imaging is indispensable for the technical success of TACE [10]. However, a longer procedure time may increase radiation exposure and contrast material use [11].

In our practice, we have successfully used our guidewire-less “*W*ireless *A*ngiographic *VE*ssel *S*election” (WAVES) technique for treating unresectable HCC involving multiple tumors, and larger tumors with multiple vessel blood supply. Herein, we aim to describe the WAVES technique, and to compare the procedure time and radiation exposure between WAVES (guidewire-less) and conventional (guidewire) techniques in superselective TACE of HCC. We hypothesize that the WAVES technique is associated with significantly less radiation exposure than conventional TACE.

## Methods

### Study design and patients

This records of patients who received conventional transarterial chemoembolization (cTACE), drug-eluting chemoembolization (DEB-TACE), or hepatic arterial chemo infusion at Taichung Veterans General Hospital, Taichung, Taiwan, between January 2020 and December 2022 were retrospectively reviewed. Patients were excluded if the treatment was not completed, and/or the patient experienced severe complications unrelated to the procedure during the procedure (*n* = 1). All procedure was conducted by a single interventional radiologist with 15 years of experience. Initially, patients were divided into those treated with WAVES and those treated with the conventional method of performing TACE. The procedures were selected based on target vessel difficulty (moderate difficulty [MD] and high difficulty [HD]) that was determined by vessel size, tortuosity, and angulation during nonselective initial angiography [5]. Most MD patients received the WAVES procedure initially, except for a few who underwent the conventional procedure owing to a replaced hepatic artery, vascular variants, or multiple treatment sessions, whereas most HD patients received the conventional procedure initially, with a few undergoing WAVES due to relatively large vessel size (such as in native cases). However, among WAVES group, some patients (hybrid) were switched to conventional procedure if the desired location could not be reached after 3 min of initiated WAVES procedure. The study protocol was approved by the IRB board of Taichung Veterans General Hospital (approval number: CE21391B). The requirement for informed consent of individual patients was waived as data presented in this study are pooled and de-identified.

### Surgical procedure

**Fig. 1:**
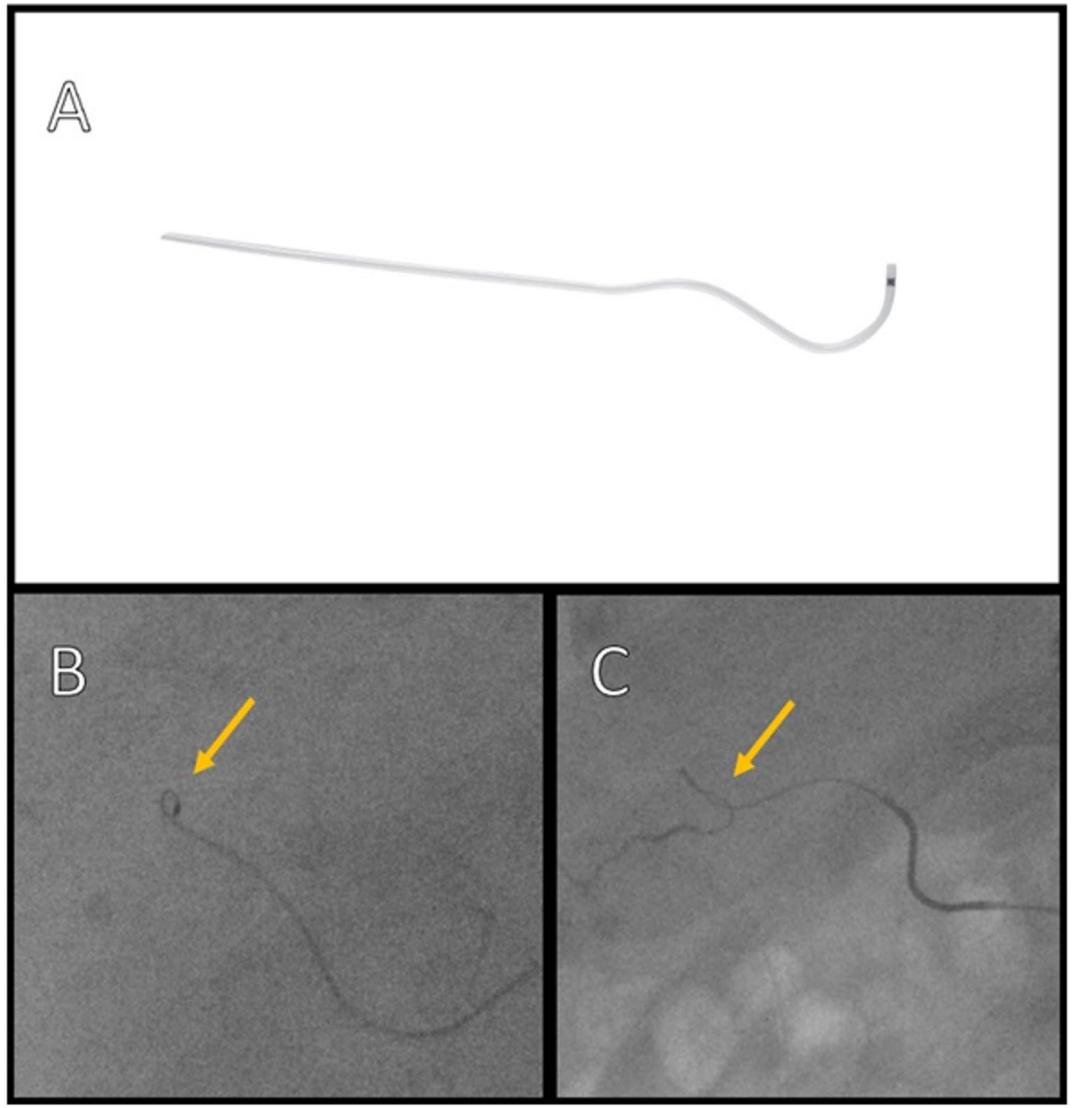
**A** Merit Maestro^®^ Microcatheter Swan neck. **B** Ensure that the microcatheter is smooth and not winding by feeling for resistance while pushing (arrow). **C** Prevent kinking due to excessive force in a 2.4-Fr Merit Maestro microcatheter (arrow)

The WAVES vessel selection/superselection procedure is largely the same as that performed with a guidewire, with the following guidelines. 1) Choose a supportive microcatheter (Maestro Swan Neck steerable microcatheter from Merit Medical [South Jordan, UT, USA]), with a soft, rounded tip, and size of 2.4 Fr to 2.1 Fr. 2) Vessel selection is easier using pre-shaped microcatheters, especially in cases where the targeted vessel branches from the parent vessel at an acute angle. 3) Use a swan neck tip microcatheter with a double-curve resembling an “S” (Fig. 1A). 4) Gently push the microcatheter into the target vessel while turning with a smooth motion. Do not push with great force or with rough, abrupt motions, as doing so may kink the tip or body of the microcatheter or damage the vessel causing intimal dissection. 5) Injecting contrast media while inserting the microcatheter may be helpful to identify the orifices of the target vessel branches. 6) Torque the microcatheter at each bifurcation of the vessel with smooth, gentle motions to ensure that it turns into the right direction. 7) Ensure that the microcatheter is smooth and not winding by feeling for resistance while pushing (Fig. 1B and C). 8) Use cone-beam computed tomography (CT) to help identify both the target vessel and area of the liver that is covering the tumor.

The conventional technique was performed with the above-mentioned Maestro Swan microcatheter in conjunction with the Transend^®^ 0.014 Shapeable Guidewire from Stryker Corporation (Kalamazoo, MI, USA).

### Data collection and evaluation

**Fig. 2:**
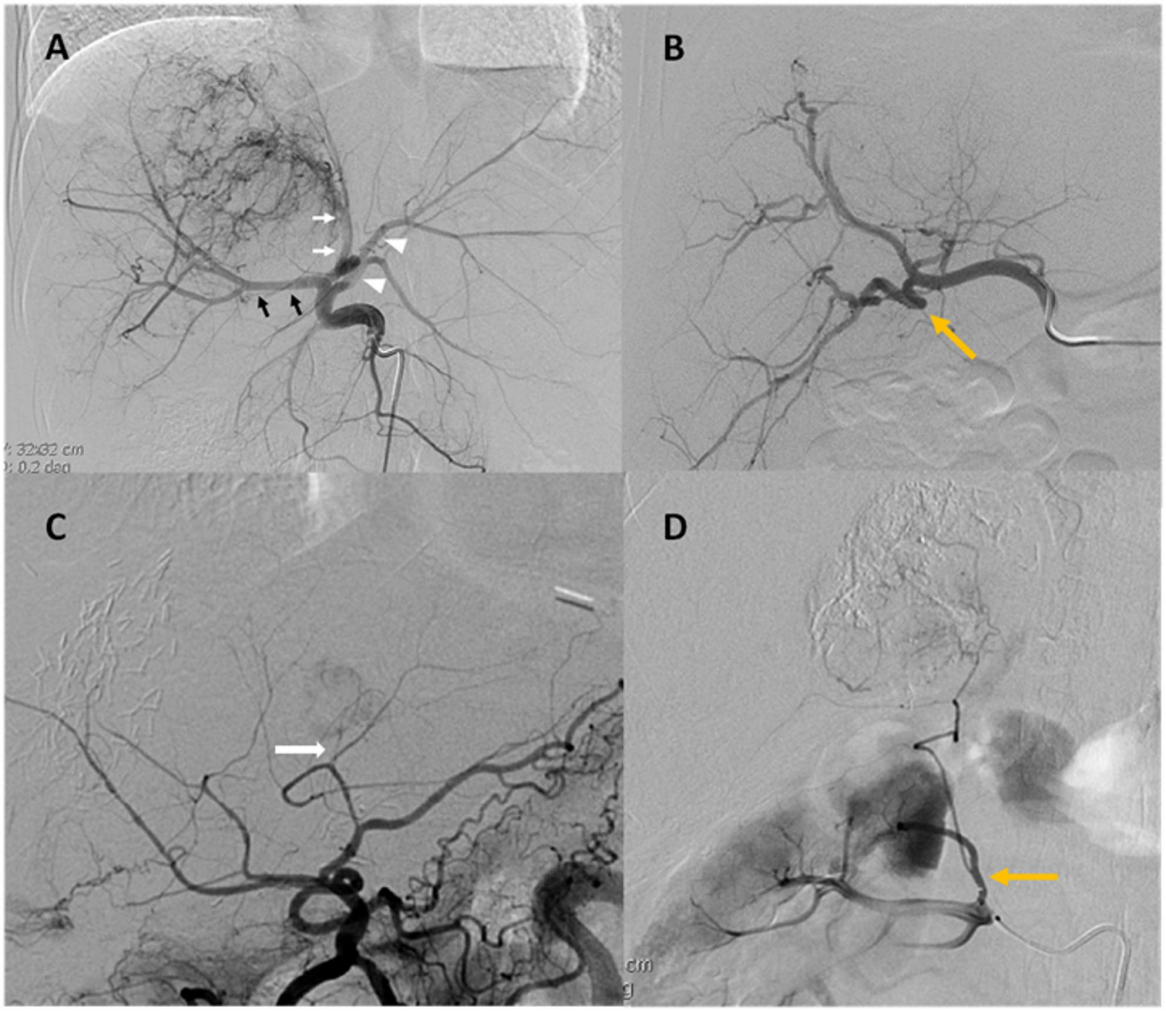
Target vessel difficulty was divided into moderate (MD) (**A)** all arrows and arrowheads and high (HD), and determined during nonselective initial angiography based on (**B**) tortuosity, (**C**) vessel size, and (**D**) angulation. HCC blood was supplied from right renal artery

Baseline demographic and clinical data collected from the medical records were age, sex, number of tumors, sizes of the tumors, treatment type, vessel type, vessel branch, number of target vessels, procedure type, procedure time, and radiation dose. The differentiation between high-difficulty (HD) and moderate-difficulty (MD) vessels was based on predefined angiographic criteria assessed at the time of the procedure, rather than on subjective post hoc evaluation. Vessel difficulty was determined using objective anatomical characteristics known to affect microcatheter navigation based on vessel caliber, degree of tortuosity, and severity of angulation, which was modified from the definition by Hoffman et al. [5] (Fig. 2). Vessel caliber was evaluated by luminal diameter on angiography relative to the microcatheter size. Vessels were classified as small caliber when the vessel diameter was less than twice the outer diameter of the selected microcatheter, and as large caliber when the vessel diameter was equal to or greater than twice the microcatheter diameter. Vessel tortuosity was defined by the degree of vessel curvature along the target segment. Tortuosity was considered low when the vessel demonstrated gentle curvature corresponding to less than a half circle or fewer than two significant bends. Tortuosity was considered high when the vessel demonstrated pronounced curvature, corresponding to a half circle or more, or two or more consecutive bends that impeded smooth microcatheter advancement. Vessel angulation referred to discrete directional changes along the vessel course and was categorized based on the most acute bend encountered. Angulation was classified as mild when bend angles were ≥ 90°, moderate when angles were between 60° and 89°, and severe when angles were < 60°. Vessels exhibiting unfavorable characteristics—small caliber, high tortuosity, and/or severe angulation—were classified as HD, while vessels with none unfavorable characteristic were classified as MD. Cases were classified according to difficulty of the vessels treated. Cases with more than one target vessel catheterized were categorized according to the vessel of highest difficulty. The overall procedural time of cTACE was defined as the time between the insertion and the removal of the angiographic sheath [12]. Following standard TACE, the celiac trunk was catheterized and digital subtraction angiography (DSA) was performed to define the target anatomy. The angio-catheter was then placed at the orifice of the celiac trunk, common hepatic artery, superior mesenteric artery (SMA), or other vasculature that had been replaced or parasitized, and the microcatheter was placed into the angio-catheter. As cone-beam CT usage varies greatly from patient to patient, and does not directly impact the physical act of selection, we subtracted it from “procedure time”, indicating procedure time difference in different procedure approach.

The primary outcomes, procedure time and radiation dose, were compared between the WAVES and conventional groups. In the per-treatment analysis, procedure time and radiation were also compared between the WAVES, hybrid and conventional groups. The technical success rate and major postoperative complication were also evaluated. Technical success was defined as successful TACE after selection/superselection of the target vessel. Selective TACE was defined as TACE at the lobar and segmental hepatic artery, whereas superselective TACE was defined as TACE at the subsegmental hepatic artery. In tumors with only one feeding vessel, superselective TACE was required. In tumors with multiple feeders, selective TACE was acceptable and the main tumor feeder was embolized last [9]. Major complications were defined as vessel perforation, dissection, and other intimal injuries.

### Statistical analysis

Continuous variables were expressed as median and range, and were compared using the Kruskal-Wallis test. Categorical variables were compared with the chi-squared test or Fisher’s exact test. Linear regression analysis was used to determine whether there were associations between patient characteristic and procedure time and radiation dose. Significant parameters in univariate linear analysis (*p* < 0.100) were entered into multivariate linear regression analysis. A *p-*value of < 0.05 was considered statistically significant. All statistical analysis was performed using SPSS version 25.0 software (IBM Corp., New York, USA).

## Results

### Patient characteristics

Demographic and clinical characteristics of patients with HCC who received cTACE, DEB-TACE, or chemo infusion are shown in Table 1. Initially, patients were divided into 2 groups: 125 cases were treated with WAVES and 59 cases were treated with a conventional procedure. The distribution of age, sex, tumor number, tumor size, and treatment type were not significantly different between the 2 groups (all, *p* > 0.05), while the distribution of vessel type, vessel branch, and number of target vessels were significantly different between the 2 group (all, *p* < 0.05). However, among WAVES group, 36 patients (hybrid) were switched to conventional procedure because the desired location could not be reached after 3 min of initiated WAVES procedure. For this aspect, patients were further divided into 3 groups (WAVES, hybrid, and conventional), and the differences between the 3 groups were compared (per-treatment analysis). 89 patients were treated with WAVES, 36 patients were treated with hybrid procedure, and 59 patients were treated with a conventional procedure (Table 1). Similarly, the distribution of age, sex, tumor number, tumor size, and treatment type were not significantly different between the 3 groups (all, *p* > 0.05), while the distribution of vessel type, vessel branch, and number of target vessels were significantly different between the 3 group (all, *p* < 0.05).

**Table 1:** Patient demographic and clinical characteristics

Variables	WAVES(n=125)	Conventional(n=59)	*p*-value
Age (years), median (range)	69 (15–92)	71 (33–83)	0.152
Gender, n (%)			1.000
Male	88 (70.4)	42 (71.2)	
Female	37 (29.6)	17 (28.8)	
Number of tumor, n (%)			0.155
<4	68 (54.4)	25 (42.4)	
≧4 (multiple)	57 (45.6)	34 (57.6)	
Tumor size (cm), median (range)	2.8 (0.3–19.8.3.8)	2.6 (1.0–16.7.0.7)	0.721
Treatment type, n (%)			0.070
cTACE	82 (65.6)	39 (66.1)	
DEB-TACE	30 (24.0)	19 (32.2)	
Chemo infusion	13 (10.4)	1 (1.7)	
Vessel type, n (%)			<0.001*
Moderate difficulty (MD)	79 (63.2)	12 (20.3)	
High difficulty (HD)	46 (36.8)	47 (79.7)	
Vessel branch			0.004*
Lobar	6 (4.8)	0 (0)	
Segment	81 (64.8)	27 (45.8)	
Subsegment	38 (30.4)	32 (54.2)	
Number of target vessel, n (%)			<0.001*
1	79 (63.2)	20 (33.9)	
2	38 (30.4)	29 (49.2)	
3	8 (6.4)	8 (13.6)	
4	0 (0)	2 (3.4)	
Treatment time (min), median (range)	28 (10–85)	50 (11–92)	<0.001*
MD	26 (10–55)	49 (19–92)	<0.001*
HD	34 (15–85)	50 (11–91)	<0.001*
Radiation Dose (µGym^2^), median (range)	478 (102–1177)	798 (178–1745)	<0.001*

*WAVES* wireless angiographic vessel selection, *cTACE* Conventional transarterial chemoembolization, *DEB-TACE* Drug-eluting bead transarterial chemoembolization

*p<0.05

### Outcomes of the 2 groups

**Fig. 3:**
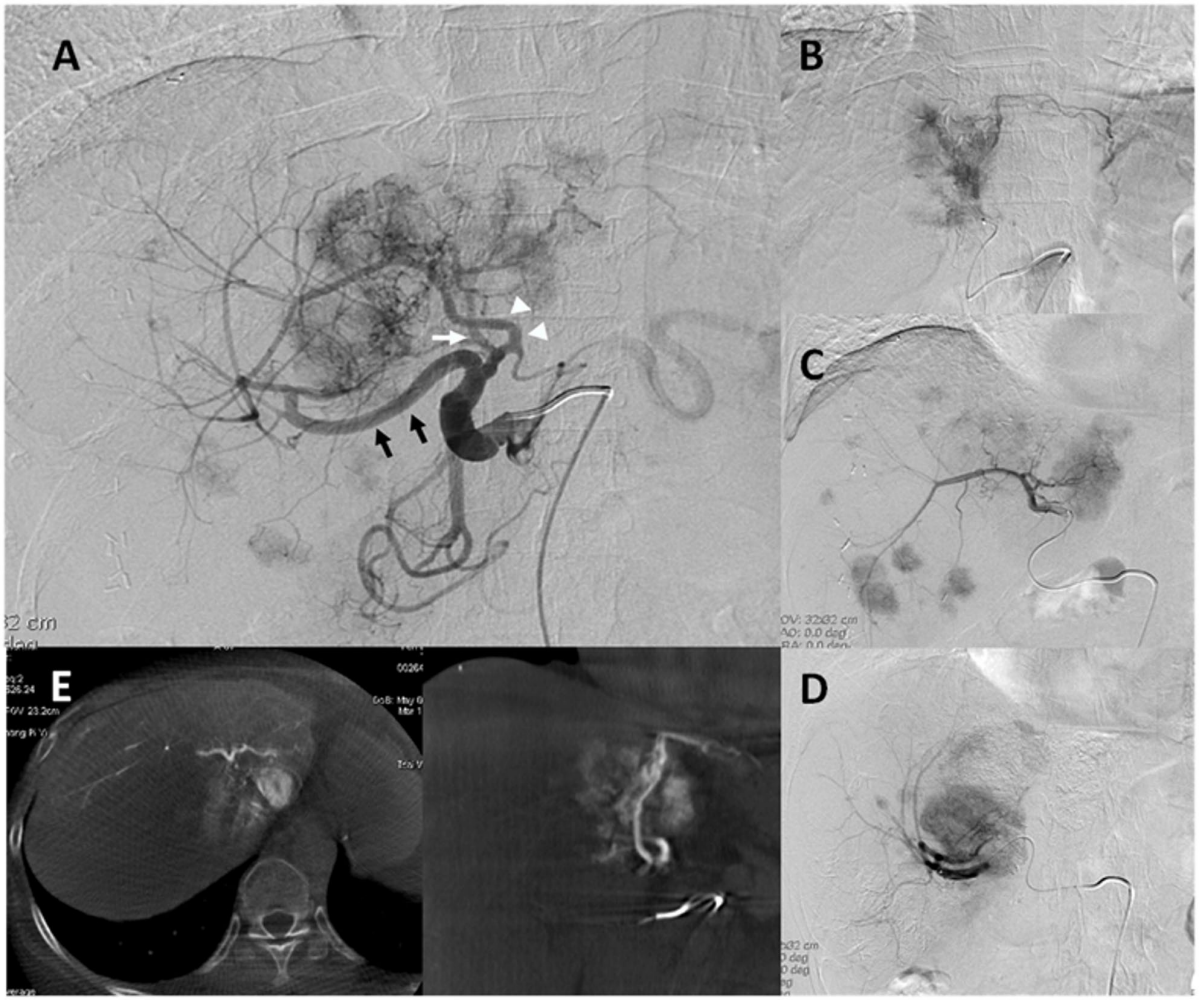
A “typical” WAVES case. A 65-year-old female with sigmoid colon cancer with liver metastases received TACE via WAVES. Multiple hypervascular tumor stains were noted on both lobes of liver, and blood was supplied by the left hepatic artery (LHA), middle hepatic artery (MHA), and right hepatic artery (RHA). **A** Superselection of the LHA, MHA, and RHA was accomplished by using the WAVES technique with 2.4-Fr. Merit Maestro microcatheter (**B**-**D**). Cone-beam CT was used to help identify both the target vessels and area of the liver that was covering the tumor(s) (**E**)

Technical success was achieved in 100% (59/59) of cases with conventional procedure, technical success was achieved in 71% (89/125) of cases with initial WAVES, and 29% (36/125) of cases were switched to conventional procedure when the desired location was not reached after 3 min of initial WAVES (hybrid group). No major related complications arose during any of the 3 procedures. A typical WAVES case in a 65-year-old female with sigmoid colon cancer with liver metastases who received TACE via WAVES is shown in Fig. 3.

Compared to the conventional group, WAVES group (28 vs. 50 min) and their MD (26 vs. 49 min)/HD (34 vs. 50 min) subgroups had a shorter procedure time, and WAVES group had a lower radiation dose (478 vs. 798 µGym^2^) (all, *p* < 0.05, Table 1). In the per-treatment analysis (Table S1), WAVES/hybrid groups (27 vs. 33.5 vs. 50 min) and their MD (26 vs. 26 vs. 49 min)/HD (34 vs. 35 vs. 50 min) subgroups had a shorter procedure time, and WAVES/hybrid groups had a lower radiation dose (455 vs. 556.5 vs. 798 µGym^2^) (all, *p* < 0.05).

### Predictors of procedure time

In the univariate linear regression analysis, procedure time-related factors, including vessel type, vessel branch, number of target vessels, and procedure type were identified by univariate linear regression analysis (all, *p* < 0.100, Table 2). After adjusted for these variables, multivariate linear regression analysis showed that WAVES procedure (β = −13.694, 95% confidence interval [CI]: −18.215, −9.173, *p* < 0.001) was an independent predictor of shorter procedure time as compared to the conventional procedure (Table 2). However, the vessel type, vessel branch, and number of target vessels were not significantly associated with procedure time (all, *p* > 0.05, Table 2). A similar observation was also found in the per-treatment analysis (Table S2). In the univariate linear regression analysis, procedure time-related factors, including vessel type, vessel branch, number of target vessels, and procedure type were identified by univariate linear regression analysis (all, *p* < 0.100). After adjusted for these variables, multivariate linear regression analysis showed that WAVES (β = −15.883, 95% confidence interval [CI]: −20.990, −10.776, *p* < 0.001) or hybrid (β = −12.202, 95% confidence interval [CI]: −17.697, −6.708, *p* < 0.001) procedure was an independent predictor of shorter procedure time as compared to the conventional procedure. However, the vessel type, vessel branch, and number of target vessels were not significantly associated with procedure time (all, *p* > 0.05).

**Table 2:** Univariate and multivariate linear regression analysis of procedure time and predictors

Predictors	Univariate		Multivariate	
	β (95% CI)	p-value	β (95% CI)	*p*-value
Age (years)	0.031 (−0.192, 0.253)	0.786		
Sex				
Female	Reference			
Male	−1.077 (−6.254, 4.101)	0.682		
Number of tumor				
< 4	Reference			
≥ 4	1.158 (−3.557, 5.873)	0.629		
Tumor size (cm)	0.298 (−0.341, 0.937)	0.359		
Treatment				
cTACE	6.470 (−2.400, 15.341)	0.152	0.507 (−7.545, 8.559)	0.901
DEB-TACE	12.092 (2.569, 21.615)	0.013*	5.354 (−3.251, 13.958)	0.221
Chemo infusion	Reference		Reference	
Vessel type				
MD	Reference		Reference	
HD	12.409 (8.054, 16.763)	<0.001*	4.591 (−1.062, 9.243)	0.153
Vessel branch				
Lobar	−19.457 (−32.669, −6.245)	0.004*	−10.260 (−22.876, 2.356)	0.110
Segment	−6.068 (−10.834, −1.302)	0.013*	−0.520 (−4.754, 3.714)	0.809
Subsegment	Reference		Reference	
Number of target vessels				
1	−19.146 (−40.219, 1.926)	0.075	−3.418 (−21.893, 15.057)	0.715
2	−6.888 (−28.060, 14.284)	0.522	4.707 (−13.628, 23.042)	0.613
3	−4.937 (−27.066, 17.191)	0.660	3.110 (−16.001, 22.221)	0.748
4	Reference		Reference	
Procedure type				
WAVES	−18.802 (−23.042, −14.562)	<0.001*	−13.694 (−18.215, −9.173)	<0.001*
Conventional	Reference		Reference	

*WAVES* Wireless Angiographic VEssel Selection, *cTACE* Conventional transarterial chemoembolization, *DEB-TACE* Drug-eluting bead transarterial chemoembolization

*p<0.05

After stratifying for vessel type (MD and HD), an association between WAVES and shorter procedure time was also found in both MD (WAVES vs. conventional: β = −21.085, 95% CI: −28.799, −13,371, *p* < 0.001) and HD (WAVES vs. conventional: β = −10.784, 95% CI: −16.644, −4.924, *p* < 0.001) subgroups (Table S3), indicating no synergistic effect between target vessel difficulty type and WAVES procedure for shorter procedure time. A similar observation was also found in the per-treatment analysis (Table S4), indicating no synergistic effect between target vessel difficulty type and WAVES procedure for shorter procedure time. In contrast. an association between hybrid procedure and shorter procedure time was only found in the MD subgroup (hybrid vs. conventional: β = −19.771, 95% CI: −34.280, −5.263, *p* = 0.009), but this association was not observed in the HD subgroup (hybrid vs. conventional: β = −6.595, 95% CI: −15.894, 2.705, *p* = 0.160) (Table S4), indicating a synergistic effect of MD and hybrid procedure for shorter procedure time.

### Predictors of radiation dose

Radiation dose-related factors, including treatment type, vessel type, vessel branch, number of target vessels, procedure type, and procedure time were identified by univariate linear regression analysis (all, *p* < 0.100, Table 3). After adjusted for these variables, multivariate linear regression analysis showed that a longer procedure time was an independent predictor of higher radiation dose (β = 14.216, 95% CI: 12.654, 15.777, *p* < 0.001, Table 3). While a low number of target vessels was associated with a lower radiation dose (β = −224.100, 95% CI: −460.610, 12.411, *p* = 0.063, Table 3), although it was not statistically different. However, the treatment type, vessel type, vessel branch, and procedure type were not independent predictors of radiation dose (all, *p* > 0.05, Table 3). A similar observation was also found in the per-treatment analysis (Table S5). Multivariate linear regression analysis showed that a longer procedure time was an independent predictor of higher radiation dose (β = 14.389, 95% CI: 12.962, 15.816, *p* < 0.001, Table S5). While a low number of target vessels was associated with a lower radiation dose (β = −223.762, 95% CI: −461.388, 13.863, *p* = 0.065, Table S5), although it was not statistically different. However, the treatment type, vessel type, vessel branch, and procedure type were not independent predictors of radiation dose (all, *p* > 0.05, Table S5).

**Table 3:** Univariate and multivariate linear regression analysis of radiation dose and predictors

Predictors	Univariate		Multivariate	
	β (95% CI)	*p*-value	β (95% CI)	*p*-value
Age (years)	−0371. (−4.214, 3.473)	0.849		
Sex				
Female	Reference			
Male	−16.383 (−105.768, 73.002)	0.718		
Number of tumors				
< 4	Reference			
≥ 4	15.896 (−65.509, 97.301)	0.700		
Tumor size (cm)	5.914 (−5.110, 16.937)	0.291		
Treatment				
cTACE	94.463 (−58.996, 247.922)	0.226	−7.894 (−106.117, 90.329)	0.874
DEB-TACE	191.551 (26.814, 356.288)	0.023*	−13.757 (−118.158, 90.645)	0.795
Chemo infusion	Reference		Reference	
Vessel type				
MD	Reference		Reference	
HD	190.824 (114.318, 267.330)	<0.001*	−18.183 (−83.974, 47.608)	0.585
Vessel branch				
Lobar	−304.238 (−532.384, −76.092)	0.009*	−17.134 (−177.967, 143.699)	0.833
Segment	−115.886 (−198.182, −33.590)	0.006*	−21.581 (−76.067, 32.905)	0.434
Subsegment	Reference		Reference	
Number of target vessel				
1	−430.732 (−792.471, −68.993)	0.020*	−224.100 (−460.610, 12.411)	0.063
2	−220.306 (−583.752, 143.140)	0.233	−174.866 (−407.056, 57.324)	0.139
3	−177.062 (−556.927, 202.802)	0.359	−158.599 (−406.079, 88.882)	0.207
4	Reference		Reference	
Procedure type				
WAVES	−299.348 (−374.804, −223.891)	<0.001*	−33.362 (−100.777, 34.054)	0.329
Conventional	Reference		Reference	
Procedure time (min)	15.004 (13.755, 16.252)	<0.001*	14.216 (12.654, 15.777)	<0.001*

*WAVES* Wireless angiographic vessel selection, *cTACE* Conventional transarterial chemoembolization, *DEB-TACE* Drug-eluting bead transarterial chemoembolization

*p<0.05

After stratifying for vessel type (MD and HD), an association between longer procedure time and higher radiation dose was also found in both MD (β = 14.938, 95% CI: 12.694, 17.183, *p* < 0.001) and HD (β = 13.617, 95% CI: 11.293, 15.942, *p* < 0.001) subgroups (Table S6), indicating no synergistic effect between target vessel difficulty type and procedure time for higher radiation dose. After stratifying for procedure type (WAVES and conventional), multivariate linear regression analysis showed an association between longer procedure time and higher radiation dose in the WAVES (β = 14.772, 95% CI: 12.828, 16.716, *p* < 0.001) and conventional (β = 13.364, 95% CI: 10.457, 16.271, *p* < 0.001) subgroups (Table S7), indicating no synergistic effect between procedure type and procedure time for higher radiation dose. A similar observation was also found in the per-treatment analysis, indicating no synergistic effect between target vessel difficulty type and procedure time for higher radiation dose (Table S8) and no synergistic effect between procedure type (WAVES, hybrid, and conventional) and procedure time for higher radiation dose (Table S9).

## Discussion

The objective of the present study was to determine differences of procedure time and radiation exposure between superselective transarterial microcatheter procedures with WAVES (guidewire-less) and conventional (guidewire) techniques in patients with HCC receiving cTACE, DEB-TACE, or hepatic arterial chemo infusion. There are 3 new key findings of the study. (1) WAVES procedure is an independent predictor of shorter procedure time as compared to the conventional procedure in patients with MD or HD. (2) In the per-treatment analysis, WAVES or hybrid procedure is an independent predictor of shorter procedure time, there is a synergistic effect between vessel with MD and hybrid procedure for shorter procedure time. (3) A longer procedure time is an independent predictor of higher radiation dose in patients with any vessel difficulty (MD or HD) or those treated with any procedure (WAVES, hybrid or conventional procedure).

In a guidewire-less technique study, Hoffman et al. reported successful cannulations in 11 out of 20 patients in 33 out of 46 whole body vessels [5]. Although the procedure time for the 46 vessels in the steerable microcatheter group was found to be significantly lower than for the 34 vessels in the conventional microcatheter group, the study encompassed 17 considerably different target vessels and largely unspecified treatments, making it difficult to generalize their conclusions. The present study had a larger sample size (151 cases vs. 46), and focused on liver-tumor-supplying arteries of HCC patients receiving cTACE, DEB-TACE, or hepatic arterial chemo infusion; indicating that our WAVES vs. conventional comparisons are more direct with respect to the tumor-related arteries. Overall, the WAVES procedure took approximately 44% less time (median 28 min vs. 50 min) than the conventional procedure, respectively. After adjusting for other factors (treatment type, vessel type, vessel branch, and number of target vessels), the WASVES procedure was an independent predictor of shorter procedure time when compared with the conventional procedure. To determine the impact of vessel difficulty on WAVES suitability, the procedure time was further broken down by difficulty, and a similar observation was seen in the MD and HD subgroups. Furthermore, in the per-treatment analysis, above findings also observed in patients with hybrid procedure. This suggests that the WAVES procedure may be a potential option for achieving a shorter procedure time in patients with MD or HD, even though it intraoperatively switched to conventional procedure.

With regard to the impact of procedure type on radiation exposure, our data showed that the WAVES or hybrid procedure was not an independent predictor of radiation dose in patients with MD or HD, although the WAVES procedure resulted in a significantly lower radiation dose compared to the conventional procedure (median 478 µGym^2^ vs. 798 µGym^2^). In contrast, the procedure time was an independent predictor of higher radiation dose. Furthermore, in the per-treatment analysis, above findings also observed in patients with hybrid procedure. Since the identification of tumor feeders through intraprocedural imaging is crucial for the success of TACE [10], an extended procedure duration could lead to higher radiation exposure and increased use of contrast material [11]. According to these findings, we thus hypothesize that the shorter time required for the WAVES or hybrid procedure would naturally result in less radiation exposure, due to fewer opportunities for the detection of tumor feeders using intraprocedural imaging.

In this study, 36 (29%) cases received a hybrid procedure (patients were switched to conventional procedure when the desired location could not be reached after 3 min of initiated WAVES procedure), with 75% of cases with HD vessels and 25% of cases with MD vessels. This indicates that WAVES is more suitable as a first-line procedure for MD rather than HD vessels. Nevertheless, in the per-treatment analysis, the multivariate linear regression analysis showed the hybrid procedure was an independent predictor of shorter procedure time. After stratifying by vessel type (MD and HD), the hybrid procedure was an independent predictor of shorter procedure time in the MD subgroup as compared to the conventional procedure, while the hybrid procedure was not an independent predictor of procedure time in the HD subgroup. Furthermore, after stratifying by vessel type (MD and HD), a longer procedure time was an independent predictor of higher radiation dose in both MD and HD subgroups. These results suggest that most of the time savings associated with WAVES is accomplished in the earlier stages of super-selection in patients with MD or HD, particularly in the MD subgroup, with the guidewire adding an insignificant amount of time to the latter part of the procedures. In addition, the shorter WAVES or hybrid procedure time would also lead to less radiation exposure. This in turn suggests the WAVES procedure could be reasonably deployed as a first-line procedure for MD vessel, even if it needed to be intraoperatively converted to a conventional procedure.

No major procedure-related complications were observed in the WAVES group, including vessel perforation and intimal injury, possibly because the lack of a guidewire appears to be at least as safe as conventional techniques [13, 14]. In 2019, Keuler et al. reported on endovascular thrombectomies and suggested that it was significantly safer to pass clots using wireless microcatheter techniques [7]. The following rationales for WAVES as a first-line approach for inoperable HCC was arrived at by combining the results of our study with those of Keuler et al. (1) Guidewire-less techniques such as WAVES are at least as safe as conventional techniques with guidewires with regards to intimal injury, vessel perforation, and similar complications. (2) For MD or HD vessels, the WAVES or hybrid procedure is significantly associated with a shorter procedure time, resulting in a lower radiation exposure, suggesting that WAVES should always be deployed first.

The radiology practice model was significantly impacted by the COVID-19 pandemic. In March 2020 alone, a majority of practices globally witnessed an unparalleled reduction in their volume of greater than 50% [15, 16]. Even in countries where the hospitalization rate for the novel coronavirus was comparatively low, significant adjustments were necessary in patient management to mitigate the transmission risk, particularly among vulnerable populations like oncology patients [17]. As a result of the COVID-19 pandemic, the timing aspect has been underscored than ever, emphasizing the critical need for meticulous time management and scheduling to ensure the optimal efficiency of procedures and treatments while still maintaining the patient’s safety with the proper distancing, as imposed by the pandemic [17]. With respect to this, the results of the present study provide evidence supporting the potential benefit of the WAVES procedure in HCC patients receiving cTACE, DEB-TACE, or hepatic arterial chemo infusion as compared with conventional TACE. Moreover, the goal of previous studies was to improve the early diagnosis of HCC with different strategies [18, 19]; therefore, progressively less extended lesions should be treated in the future, thereby duration of treatments such as cTACE would be further reduced, decreasing the risks of radiation exposure to which both patients and interventional radiologists are exposed.

Another interesting result that emerged from our study is that the tumor size, number of tumors, treatment type, vessel type, vessel branch, and target vessel did not affect the procedure duration of the WAVES technique and conventional guidewire TACE. This result probably can be explained by the fact that the increase in the tumor size, number of tumors, vessel branch, number of target vessels, and vessel difficulty and the different treatment type does not entail particular changes in the time required for the effective administration of the chemoembolizing agent. Although catheterization of the artery using conventional guidewire TACE actually increases the procedure time when compared with the WAVES technique.

In clinical practice, in addition to safety of WAVES in HCC patients, the use of WAVES could also reduce procedure time, save the cost of one or two guidewires, reduce nursing staff costs, and reduce medical waste when compared to those with conventional procedure, which is consistent with contemporary ESG concepts. Overall, it suggests that WAVES results in decreased costs in HCC patients treated with TACE.

The main limitations of our study are due to its retrospective nature, and that all cases were from a single center and the sample size was relatively small. Therefore, we cannot establish a cause-and-effect relation between procedure type and procedure time and radiation exposure; longitudinal studies are needed to elucidate any such association. In addition, in conjunction with excluded data and the radiologist’s selection preference, there is a possibility that we may have overestimated the degree to which the WAVES method reduced procedure times, although hybrid procedure group has been compared to conventional procedure group using multivariate linear regression analysis (adjusted for these confounding factors). Procedure time excluded cone-beam CT time because that cone-beam CT usage varies greatly from patient to patient and does not directly impact the physical act of selection. However, we compared the number of patients and number of cone-beam CT usages between the WAVES and conventional groups and found no significant differences between them (*p* = 0.430).

## Conclusion

WAVES is suitable for the treatment of HCC by cTACE, DEB-TACE, or hepatic arterial chemo infusion in patients with MD or HD vessels. WAVES is associated with a shorter procedure time, which reduces radiation dose. We suggest that it may be deployed as a first-line approach for MD rather than HD vessels.

## Supplementary Information

Supplementary Material 1.

## Data Availability

The data used to support the findings of this study are included within the article and its supplementary information files.

## Abbreviations

HCC: Hepatocellular carcinoma
TACE: Transcatheter arterial chemoembolization
WAVES: Wireless Angiographic VEssel Selection
MD: Moderate target vessel difficulty
HD: High target vessel difficulty
DSA: Digital subtraction angiography
