# Supplementary material for: Efficiency and safety of a guidewire-less approach to superselective transarterial microcatheter procedures in hepatic cancer interventions
Source: BMC Cancer. 2026 Mar 25;26:558. doi: 10.1186/s12885-026-15907-5 (PMC13137538; doi:10.1186/s12885-026-15907-5)
Supplement: Supplementary file 1 — Supplementary Material 1. [file 12885_2026_15907_MOESM1_ESM.docx]

**Table S1. Patient demographic and clinical characteristics (per-treatment analysis)**

| Variables | WAVES  (n=89) | Hybrid  (n=36) | Conventional  (n=59) | p-value |
| --- | --- | --- | --- | --- |
| **Age (years)**, median (range) | 69 (15-92) | 69 (15-81) | 71 (33-83) | 0.350 |
| **Sex**, n (%) |  |  |  | 1.000 |
| Male | 63 (70.8) | 25 (69.4) | 42 (71.2) |  |
| Female | 26 (29.2) | 11 (30.6) | 17 (28.8) |  |
| **Number of tumor**, n (%) |  |  |  | 0.320 |
| <4 | 48 (53.9) | 20 (55.6) | 25 (42.4) |  |
| ≧4 (multiple) | 41 (46.1) | 16 (44.4) | 34 (57.6) |  |
| **Tumor size (cm)**, median (range) | 3.0 (0.3-19.8) | 2.75 (1.00-13.00) | 2.6 (1.0-16.7) | 0.742 |
| **Treatment type**, n (%) |  |  |  | 0.134 |
| cTACE | 61 (68.5) | 21 (58.3) | 39 (66.1) |  |
| DEB-TACE | 20 (22.5) | 10 (27.8) | 19 (32.2) |  |
| Chemo infusion | 8 (9.0) | 5 (13.9) | 1 (1.7) |  |
| **Vessel type**, n (%) |  |  |  | <0.001* |
| Moderate difficulty (MD) | 70 (78.7) | 9 (25.0) | 12 (20.3) |  |
| High difficulty (HD) | 19 (21.3) | 27 (75.0) | 47 (79.7) |  |
| **Vessel branch** |  |  |  | 0.002* |
| Lobar | 6 (6.7) | 0 (0) | 0 (0) |  |
| Segment | 60 (67.4) | 21 (58.3) | 27 (45.8) |  |
| Subsegment | 23 (25.8) | 15 (41.7) | 32 (54.2) |  |
| **Number of target vessel**, n (%) |  |  |  | 0.001* |
| 1 | 58 (65.2) | 21 (58.3) | 20 (33.9) |  |
| 2 | 28 (31.5) | 10 (27.8) | 29 (49.2) |  |
| 3 | 3 (3.4) | 5 (13.9) | 8 (13.6) |  |
| 4 | 0 (0) | 0 (0) | 2 (3.4) |  |
| **Treatment time (min)**, median (range) | 27 (10-64) | 33.5 (15-85) | 50 (11-92) | <0.001* |
| MD | 26 (10-55) | 26 (15-48) | 49 (19-92) | <0.001* |
| HD | 34 (15-64) | 35 (16-85) | 50 (11-91) | <0.001* |
| **Radiation Dose (µGym^2^)**, median (range) | 455 (102-1177) | 556.5 (203-988) | 798 (178-1745) | <0.001* |

WAVES, Wireless Angiographic VEssel Selection; cTACE, conventional transarterial chemoembolization; DEB-TACE, drug-eluting bead transarterial chemoembolization. *p<0.05.

**Table S2. Univariate and multivariate linear regression analysis of procedure time and predictors (per-treatment analysis)**

| **Predictors** | **Univariate** | |  | **Multivariate** | |
| --- | --- | --- | --- | --- | --- |
|  | **β (95% CI)** | **p-value** |  | **β (95% CI)** | **p-value** |
| **Age (years)** | 0.031 (-0.192, 0.253) | 0.786 |  |  |  |
| **Sex** |  |  |  |  |  |
| Female | Reference |  |  |  |  |
| Male | -1.077 (-6.254, 4.101) | 0.682 |  |  |  |
| **Number of tumor** |  |  |  |  |  |
| < 4 | Reference |  |  |  |  |
| ≥ 4 | 1.158 (-3.557, 5.873) | 0.629 |  |  |  |
| **Tumor size (cm)** | 0.298 (-0.341, 0.937) | 0.359 |  |  |  |
| **Treatment** |  |  |  |  |  |
| cTACE | 6.470 (-2.400, 15.341) | 0.152 |  | 0.988 (-7.111, 9.087) | 0.810 |
| DEB-TACE | 12.092 (2.569, 21.615) | 0.013* |  | 5.518 (-3.089, 14.125) | 0.207 |
| Chemo infusion | Reference |  |  | Reference |  |
| **Vessel type** |  |  |  |  |  |
| MD | Reference |  |  | Reference |  |
| HD | 12.409 (8.054, 16.763) | <0.001* |  | 2.316 (-2.642, 7.273) | 0.358 |
| **Vessel branch** |  |  |  |  |  |
| Lobar | -19.457 (-32.669, -6.245) | 0.004* |  | -11.038 (-22.419, 0.343) | 0.057 |
| Segment | -6.068 (-10.834, -1.302) | 0.013* |  | -0.833 (-4.996, 3.331) | 0.694 |
| Subsegment | Reference |  |  | Reference |  |
| **Number of target vessels** |  |  |  |  |  |
| 1 | -19.146 (-40.219, 1.926) | 0.075 |  | -4.986 (-23.559, 13.586) | 0.597 |
| 2 | -6.888 (-28.060, 14.284) | 0.522 |  | 3.598 (-14.842, 22.038) | 0.701 |
| 3 | -4.937 (-27.066, 17.191) | 0.660 |  | 2.270 (-16.967, 21.508) | 0.816 |
| 4 | Reference |  |  | Reference |  |
| **Procedure type** |  |  |  |  |  |
| WAVES | -20.584 (-25.037, -16.132) | <0.001* |  | -15.883 (-20.990, -10.776) | <0.001* |
| Hybrid | -14.395 (-20.004, -8.786) | <0.001* |  | -12.202 (-17.697, -6.708) | <0.001* |
| Conventional | Reference |  |  | Reference |  |

WAVES, Wireless Angiographic VEssel Selection; cTACE, conventional transarterial chemoembolization; DEB-TACE, drug-eluting bead transarterial chemoembolization. *p<0.05.

**Table S3. Multivariate linear regression analysis of procedure time and predictors after stratifying for vessel type**

| **Predictors** | **MD (n=91)** | |  | **HD (n=93)** | |
| --- | --- | --- | --- | --- | --- |
|  | **β (95% CI)** | **p-value** |  | **β (95% CI)** | **p-value** |
| **Treatment** |  |  |  |  |  |
| cTACE | 3.190 (-8.546, 14.925) | 0.590 |  | -1.415 (-12.804, 9.974) | 0.805 |
| DEB-TACE | 4.294 (-7.810, 16.398) | 0.482 |  | 5.791 (-6.748, 18.330) | 0.361 |
| Chemo infusion | Reference |  |  | Reference |  |
| **Vessel branch** |  |  |  |  |  |
| Lobar | -5.674 (-19.263, 7.914) | 0.409 |  | － |  |
| Segment | 2.210 (-3.946, 8.366) | 0.477 |  | -2.480 (-8.451, 3.491) | 0.411 |
| Subsegment | Reference |  |  | Reference |  |
| **Number of target vessels** |  |  |  |  |  |
| 1 | -7.383 (-13.035, 1.731) | 0.111 |  | -4.174 (-24.212, 15.864) | 0.680 |
| 2 | Reference |  |  | 5.308 (-14.462, 25.079) | 0.595 |
| 3 | － |  |  | 2.957 (-17.544, 23.458) | 0.775 |
| 4 | － |  |  | Reference |  |
| **Procedure type** |  |  |  |  |  |
| WAVES | -21.085 (-28.799, -13.371) | <0.001* |  | -10.784 (-16.644, -4.924) | <0.001* |
| Conventional | Reference |  |  | Reference |  |

WAVES, Wireless Angiographic VEssel Selection; cTACE, conventional transarterial chemoembolization; DEB-TACE, drug-eluting bead transarterial chemoembolization. *p<0.05.

**Table S4. Multivariate linear regression analysis of procedure time and predictors after stratifying for vessel type (per-treatment analysis)**

| **Predictors** | **MD (n=91)** | |  | **HD (n=93)** | |
| --- | --- | --- | --- | --- | --- |
|  | **β (95% CI)** | **p-value** |  | **β (95% CI)** | **p-value** |
| **Treatment** |  |  |  |  |  |
| cTACE | 23.606 (-2.114, 49.326) | 0.071 |  | -2.310 (-35.799, 31.179) | 0.890 |
| DEB-TACE | 22.420 (-5.500, 50.340) | 0.112 |  | 0.743 (-33.916, 35.402) | 0.966 |
| Chemo infusion | Reference |  |  | Reference |  |
| **Vessel branch** |  |  |  |  |  |
| Lobar | 24.911 (-10.218, 60.040) | 0.158 |  | － |  |
| Segment | 4.348 (-4.805, 13.501) | 0.340 |  | -3.641 (-11.767, 4.486) | 0.371 |
| Subsegment | Reference |  |  | Reference |  |
| **Number of target vessels** |  |  |  |  |  |
| 1 | -3.990 (-14.762, 6.783) | 0.456 |  | 5.011 (-35.920, 45.942) | 0.806 |
| 2 | Reference |  |  | 22.144 (-20.318, 64.605) | 0.299 |
| 3 | － |  |  | 14.742 (-28.181, 57.666) | 0.492 |
| 4 | － |  |  | Reference |  |
| **Procedure type** |  |  |  |  |  |
| WAVES | -23.167 (-34.008, -12.326) | <0.001* |  | -17.318 (-26.849, -7.787) | 0.001* |
| Hybrid | -19.771 (-34.280, -5.263) | 0.009* |  | -6.595 (-15.894, 2.705) | 0.160 |
| Conventional | Reference |  |  | Reference |  |

WAVES, Wireless Angiographic VEssel Selection; cTACE, conventional transarterial chemoembolization; DEB-TACE, drug-eluting bead transarterial chemoembolization. *p<0.05.

**Table S5. Univariate and multivariate linear regression analysis of radiation dose and predictors (per-treatment analysis)**

| **Predictors** | **Univariate** | |  | **Multivariate** | |
| --- | --- | --- | --- | --- | --- |
|  | **β (95% CI)** | **p-value** |  | **β (95% CI)** | **p-value** |
| **Age (years)** | -0371. (-4.214, 3.473) | 0.849 |  |  |  |
| **Sex** |  |  |  |  |  |
| Female | Reference |  |  |  |  |
| Male | -16.383 (-105.768, 73.002) | 0.718 |  |  |  |
| **Number of tumors** |  |  |  |  |  |
| < 4 | Reference |  |  |  |  |
| **≥** 4 | 15.896 (-65.509, 97.301) | 0.700 |  |  |  |
| **Tumor size (cm)** | 5.914 (-5.110, 16.937) | 0.291 |  |  |  |
| **Treatment** |  |  |  |  |  |
| cTACE | 94.463 (-58.996, 247.922) | 0.226 |  | -8.621 (-108.244, 91.002) | 0.864 |
| DEB-TACE | 191.551 (26.814, 356.288) | 0.023* |  | -14.329 (-119.753, 91.095) | 0.788 |
| Chemo infusion | Reference |  |  | Reference |  |
| **Vessel type** |  |  |  |  |  |
| MD | Reference |  |  | Reference |  |
| HD | 190.824 (114.318, 267.330) | <0.001* |  | -16.806 (-87.930, 54.318) | 0.641 |
| **Vessel branch** |  |  |  |  |  |
| Lobar | -304.238 (-532.384, -76.092) | 0.009* |  | -17.339 (-178.918, 144.240) | 0.832 |
| Segment | -115.886 (-198.182, -33.590) | 0.006* |  | -21.485 (-76.238, 33.269) | 0.439 |
| Subsegment | Reference |  |  | Reference |  |
| **Number of target vessel** |  |  |  |  |  |
| 1 | -430.732 (-792.471, -68.993) | 0.020* |  | -223.762 (-461.388, 13.863) | 0.065 |
| 2 | -220.306 (-583.752, 143.140) | 0.233 |  | -174.828 (-408.028, 58.372) | 0.140 |
| 3 | -177.062 (-556.927, 202.802) | 0.359 |  | -158.632 (-407.189, 89.924) | 0.209 |
| 4 | Reference |  |  | Reference |  |
| **Procedure type** |  |  |  |  |  |
| WAVES | -331.851 (-411.036, -252.666) | <0.001* |  | -31.422 (-108.615, 45.770) | 0.422 |
| Hybrid | -218.992 (-318.743, -119.240) | <0.001* |  | -35.153 (-111.022, 40.717) | 0.361 |
| Conventional | Reference |  |  | Reference |  |
| **Procedure time (min)** | 15.004 (13.755, 16.252) | <0.001* |  | 14.389 (12.962, 15.816) | <0.001* |

WAVES, Wireless Angiographic VEssel Selection; cTACE, conventional transarterial chemoembolization; DEB-TACE, drug-eluting bead transarterial chemoembolization. *p<0.05

**Table S6. Multivariate linear regression analysis of radiation dose and predictors after stratifying for vessel type**

| **Predictors** | **MD (n=91)** | |  | **HD (n=93)** | |
| --- | --- | --- | --- | --- | --- |
|  | **β (95% CI)** | **p-value** |  | **β (95% CI)** | **p-value** |
| **Treatment** |  |  |  |  |  |
| cTACE | -36.879 (-185.666, 111.909) | 0.621 |  | 5.510 (-205.335, 216.356) | 0.958 |
| DEB-TACE | -31.418 (-182.458, 119.623) | 0.678 |  | -83.711 (-303.195, 135.772) | 0.446 |
| Chemo infusion | Reference |  |  | Reference |  |
| **Vessel branch** |  |  |  |  |  |
| Lobar | -71.137 (-244.316, 102.042) | 0.413 |  | － |  |
| Segment | -55.190 (-142.056, 31.676) | 0.208 |  | -32.266 (-120.960, 56.427) | 0.467 |
| Subsegment | Reference |  |  | Reference |  |
| **Target vessels number** |  |  |  |  |  |
| 1 | -53.421 (-126.314, 19.471) | 0.147 |  | -304.396 (-596.639, -12.154) | 0.042* |
| 2 | Reference |  |  | -257.789 (-545.368, 29.791) | 0.078 |
| 3 | － |  |  | -224.550 (-533.346, 84.247) | 0.150 |
| 4 | － |  |  | Reference |  |
| **Procedure type** |  |  |  |  |  |
| WAVES | -126.615 (-271.839, 18.609) | 0.086 |  | 5.855 (-99.304, 111.013) | 0.911 |
| Conventional | Reference |  |  | Reference |  |
| **Procedure time (min)** | 14.938 (12.694, 17.183) | <0.001* |  | 13.617 (11.293, 15.942) | <0.001* |

WAVES, Wireless Angiographic VEssel Selection; cTACE, conventional transarterial chemoembolization; DEB-TACE, drug-eluting bead transarterial chemoembolization. *p<0.05.

**Table S7. Multivariate linear regression analysis of radiation dose and predictors after stratifying for procedure type**

| **Predictors** | **WAVES** | |  | **Conventional** | |
| --- | --- | --- | --- | --- | --- |
|  | **β (95% CI)** | **p-value** |  | **β (95% CI)** | **p-value** |
| **Treatment** |  |  |  |  |  |
| cTACE | -7.307 (-95.126, 80.512) | 0.869 |  | 360.624 (-335.519, 1056.767) | 0.290 |
| DEB-TACE | -32.199 (-131.447, 67.048) | 0.520 |  | 367.951 (-318.313, 1054.215) | 0.274 |
| Chemo infusion | Reference |  |  | Reference |  |
| **Vessel type** |  |  |  |  |  |
| MD | Reference |  |  | Reference |  |
| HD | -22.720 (-40.596, 86.036) | 0.477 |  | 54.451 (-215.283, 324.185) | 0.677 |
| **Vessel branch** |  |  |  |  |  |
| Lobar | -57.412 (-114.723, -0.042) | 0.048* |  | － |  |
| Segment | -58.412 (-194.993, 78.170) | 0.397 |  | -24.341 (-166.823, 118.142) | 0.725 |
| Subsegment | Reference |  |  | Reference |  |
| **Number of target vessels** |  |  |  |  |  |
| 1 | 127.374 (-9.724, 245.024) | 0.064 |  | -328.463 (-721.518, 64.592) | 0.096 |
| 2 | Reference |  |  | -288.479 (-664.933, 87.976) | 0.125 |
| 3 | － |  |  | -113.860 (-580.767, 353.046) | 0.616 |
| 4 | － |  |  | Reference |  |
| **Procedure time min** | 14.772 (12.828, 16.716) | <0.001* |  | 13.364 (10.457, 16.271) | <0.001* |

WAVES, Wireless Angiographic VEssel Selection; cTACE, conventional transarterial chemoembolization; DEB-TACE, drug-eluting bead transarterial chemoembolization. *p<0.05.

**Table S8. Multivariate linear regression analysis of radiation dose and predictors after stratifying for vessel type (per-treatment analysis)**

| **Predictors** | **MD (n=91)** | |  | **HD (n=93)** | |
| --- | --- | --- | --- | --- | --- |
|  | **β (95% CI)** | **p-value** |  | **β (95% CI)** | **p-value** |
| **Treatment** |  |  |  |  |  |
| cTACE | -37.031 (-191.242, 117.180) | 0.631 |  | 4.697 (-210.246, 219.641) | 0.965 |
| DEB-TACE | -31.547 (-186.939, 123.845) | 0.685 |  | -84.562 (-308.326, 139.202) | 0.450 |
| Chemo infusion | Reference |  |  | Reference |  |
| **Vessel branch** |  |  |  |  |  |
| Lobar | -71.238 (-247.763, 105.288) | 0.421 |  | － |  |
| Segment | -55.167 (-143.145, 32.810) | 0.213 |  | -32.057 (-122.089, 57.975) | 0.476 |
| Subsegment | Reference |  |  | Reference |  |
| **Target vessels number** |  |  |  |  |  |
| 1 | -53.429 (-127.152, 20.294) | 0.152 |  | -303.369 (-600.948, -5.790 | 0.046* |
| 2 | Reference |  |  | -257.551 (-548.812, 33.711) | 0.082 |
| 3 | － |  |  | -224.063 (-537.073, 88.948) | 0.156 |
| 4 | － |  |  | Reference |  |
| **Procedure type** |  |  |  |  |  |
| WAVES | -126.481 (-276.319, 23.358) | 0.096 |  | 8.207 (-120.600, 137.014) | 0.898 |
| Hybrid | -126.918 (-288.443, 34.608) | 0.121 |  | 4.027 (-116.431, 124.484) | 0.947 |
| Conventional | Reference |  |  | Reference |  |
| **Procedure time (min)** | 15.046 (13.097, 16.995) | <0.001* |  | 13.869 (11.719, 16.018) | <0.001* |

WAVES, Wireless Angiographic VEssel Selection; cTACE, conventional transarterial chemoembolization; DEB-TACE, drug-eluting bead transarterial chemoembolization. *p<0.05.

**Table S9. Multivariate linear regression analysis of radiation dose and predictors after stratifying for procedure type (per-treatment analysis)**

| **Predictors** | **WAVES** | |  | **Hybrid** |  |  | **Conventional** | |
| --- | --- | --- | --- | --- | --- | --- | --- | --- |
|  | **β (95% CI)** | **p-value** |  | **β (95% CI)** | **p-value** |  | **β (95% CI)** | **p-value** |
| **Treatment** |  |  |  |  |  |  |  |  |
| cTACE | 41.742 (-78.310, 161.794) | 0.487 |  | -111.131 (-525.851, 303.588) | 0.456 |  | 360.624 (-335.519, 1056.767) | 0.290 |
| DEB-TACE | 0.045 (-129.109, 129.198) | 0.999 |  | -46.708 (-486.464, 393.048) | 0.758 |  | 367.951 (-318.313, 1054.215) | 0.274 |
| Chemo infusion | Reference |  |  | Reference |  |  | Reference |  |
| **Vessel type** |  |  |  |  |  |  |  |  |
| MD | Reference |  |  | Reference |  |  | Reference |  |
| HD | -18.176 (-105.661, 69.310) | 0.677 |  | 77.161 (-356.730, 202.409) | 0.444 |  | 54.451 (-215.283, 324.185) | 0.677 |
| **Vessel branch** |  |  |  |  |  |  |  |  |
| Lobar | 441.9 (76.888, 806.919) | 0.031* |  | － |  |  | － |  |
| Segment | -92.440 (-185.952, 1.173) | 0.061 |  | -61.606 (-199.147, 322.359) | 0.507 |  | -24.341 (-166.823, 118.142) | 0.725 |
| Subsegment | Reference |  |  | Reference |  |  | Reference |  |
| **Number of target vessels** |  |  |  |  |  |  |  |  |
| 1 | 121.723 (-15.249, 258.694) | 0.066 |  | -30.964 (-446.958, 385.031) | 0.828 |  | -328.463 (-721.518, 64.592) | 0.096 |
| 2 | Reference |  |  | -119.518 (-577.905, 338.869) | 0.468 |  | -288.479 (-664.933, 87.976) | 0.125 |
| 3 | － |  |  | Reference |  |  | -113.860 (-580.767, 353.046) | 0.616 |
| 4 | － |  |  | － |  |  | Reference |  |
| **Procedure time, min** | 16.291 (13.836, 18.746) | <0.001* |  | 10.668 (6.728, 14.608) | <0.001* |  | 13.395 (10.487, 16.303) | <0.001* |

WAVES, Wireless Angiographic VEssel Selection; cTACE, conventional transarterial chemoembolization; DEB-TACE, drug-eluting bead transarterial chemoembolization. *p<0.05.
